# Supplementary material for: Importance of the Immune Microenvironment in the Spontaneous Regression of Cervical Squamous Intraepithelial Lesions (cSIL) and Implications for Immunotherapy
Source: J Clin Med. 2022 Mar 5;11(5):1432. doi: 10.3390/jcm11051432 (PMC8910829; doi:10.3390/jcm11051432)
Supplement: Supplementary file 1 [file jcm-11-01432-s001.zip › jcm-1589260-supplementary.pdf]

Supplementary Table S1: Literature search

Search	Hits
(((("Cervical Intraepithelial Neoplasia"[Mesh]) OR ((Cervical Intraepithelial Neoplasm*[Title/Abstract]) OR (CIN1[Title/Abstract] OR CIN2[Title/Abstract] OR CIN3[Title/Abstract]))) OR (CIN[Title/Abstract]) OR (HSIL[Title/Abstract]) OR (high grade squamous intraepithelial lesion*[Title/Abstract]))) OR (((LSIL[Title/Abstract]) OR (low grade squamous intraepithelial lesion*[Title/Abstract])) OR ("Squamous Intraepithelial Lesions"[Mesh]))) AND (AND (((((immunology[Title/Abstract]) OR (immune[Title/Abstract])) OR (immune microenvironment[Title/Abstract])) OR (immunology[MeSH Terms])) OR (tumor microenvironment[MeSH Terms]))))	697
((((("Cervical Intraepithelial Neoplasia"[Mesh]) OR ((Cervical Intraepithelial Neoplasm*[Title/Abstract]) OR (CIN1[Title/Abstract] OR CIN2[Title/Abstract] OR CIN3[Title/Abstract]))) OR (CIN[Title/Abstract]) OR (HSIL[Title/Abstract]) OR (high grade squamous intraepithelial lesion*[Title/Abstract]))) OR (((LSIL[Title/Abstract]) OR (low grade squamous intraepithelial lesion*[Title/Abstract])) OR ("Squamous Intraepithelial Lesions"[Mesh]))) AND (AND (((((immunology[Title/Abstract]) OR (immune[Title/Abstract])) OR (immune microenvironment[Title/Abstract])) OR (immunology[MeSH Terms])) OR (tumor microenvironment[MeSH Terms]))))) AND (((natural history[Title/Abstract]) OR ("Natural History"[Mesh])) AND (etiology[Title/Abstract]))	47
((((("Cervical Intraepithelial Neoplasia"[Mesh]) OR ((Cervical Intraepithelial Neoplasm*[Title/Abstract]) OR (CIN1[Title/Abstract] OR CIN2[Title/Abstract] OR CIN3[Title/Abstract]))) OR (CIN[Title/Abstract]) OR (HSIL[Title/Abstract]) OR (high grade squamous intraepithelial lesion*[Title/Abstract]))) OR (((LSIL[Title/Abstract]) OR (low grade squamous intraepithelial lesion*[Title/Abstract])) OR ("Squamous Intraepithelial Lesions"[Mesh])))) AND (((("Imiquimod"[Mesh]) OR (1-Isobutyl-1H-imidazo(4,5-c)quinolin-4-amine[Title/Abstract] OR S 26308[Title/Abstract] OR S-26308[Title/Abstract] OR R 837[Title/Abstract] OR R-837 [Title/Abstract] OR R837[Title/Abstract] OR Zyclara[Title/Abstract] OR Aldara[Title/Abstract])) OR (imiquimod[Title/Abstract])))	54
((((("Cervical Intraepithelial Neoplasia"[Mesh]) OR ((Cervical Intraepithelial Neoplasm*[Title/Abstract]) OR (CIN1[Title/Abstract] OR CIN2[Title/Abstract] OR CIN3[Title/Abstract]))) OR (CIN[Title/Abstract]) OR (HSIL[Title/Abstract]) OR (high grade squamous intraepithelial lesion*[Title/Abstract])) AND (((regression[Title/Abstract]) OR (spontaneous regression[Title/Abstract])) OR ("Neoplasm Regression, Spontaneous"[Mesh]))) AND (((((immunology[Title/Abstract]) OR (immune[Title/Abstract])) OR (immune microenvironment[Title/Abstract])) OR (immunology[MeSH Terms])) OR (tumor microenvironment[MeSH Terms]))	109
